# Gestational diabetes mellitus, follow-up of future maternal risk of cardiovascular disease and the use of eHealth technologies—a scoping review

**DOI:** 10.1186/s13643-023-02343-w

**Published:** 2023-09-28

**Authors:** Bendik S. Fiskå, Aase Serine Devold Pay, Anne Cathrine Staff, Meryam Sugulle

**Affiliations:** 1 Division of Obstetrics and Gynaecology, Oslo University Hospital, Oslo, Norway; 2 Institute for Clinical Medicine, Faculty of Medicine, University of Oslo, Oslo, Norway; 3 Faculty of Health Sciences, Oslo Metropolitan University, Oslo, Norway; 4 Department of Obstetrics and Gynaecology, Bærum Hospital, Vestre Viken Hospital Trust, Bærum, Norway

**Keywords:** Gestational diabetes, Cardiovascular disease, Diabetes mellitus, eHealth, Scoping review, Postpartum

## Abstract

**Background:**

Globally, gestational diabetes mellitus complicates 1 in 6 pregnancies and increases future risk of type 2 diabetes and cardiovascular disease in the affected women. There is a lack of consensus on the optimal follow-up of these women. eHealth is emerging as a health care tool, but its practical utility and advantages over standard care in the follow-up after pregnancy complications remains to be determined. Our aim was to systematically review the existing literature on cardiovascular follow-up after gestational diabetes, the utility of eHealth technology for this purpose, and to identify research gaps.

**Methods:**

We performed a systematic scoping review following a published protocol and the Joanna Briggs methodology for studies up until May 2022. Four databases were searched: Ovid MEDLINE, Embase, Maternity and Infant Care, and Cochrane Database of Systematic Reviews. Primary research articles and systematic reviews were included in the final analyses. Two reviewers independently screened abstracts and performed full text assessment. Data was extracted using a data charting form. In all stages of the process, if consensus was not reached, a third reviewer was consulted. The findings from the data charting process provided the basis for summarizing the findings from the included studies.

**Results:**

The search of the databases generated 2772 hits. After removing duplicates and manually adding a total of 19 studies, reviews, and guidelines, a total of 2769 titles and abstracts were screened, and 97 papers underwent full-text review. In the final analyses, 15 articles and 12 systematic reviews were included, whereas guidelines are presented as supplementary material.

No studies were identified that examined follow-up regarding long-term overall cardiovascular risk after gestational diabetes. Various lifestyle interventions were tested for individual cardiovascular risk factors, with diverging effects. eHealth technologies were found acceptable by participants but had no consistent, statistically significant effect on relevant health outcomes.

**Conclusions:**

This scoping review of the existing literature revealed neither an established systematic cardiovascular follow-up strategy for women after gestational diabetes nor evidence that eHealth technologies are superior to conventional follow-up. Further research into the utility of eHealth in cardiovascular follow-up after complicated pregnancies should include longer-term follow-up and core cardiovascular outcomes.

**Systematic review registration:**

The protocol for this scoping review was published at Open Science Framework (osf.io/p5hw6)

**Supplementary Information:**

The online version contains supplementary material available at 10.1186/s13643-023-02343-w.

## Introduction

Gestational diabetes mellitus (GDM) is a common complication of pregnancy, with a rising incidence, affecting around 1 in 6 births globally, with prevalence varying across different regions and populations [1]. GDM impacts maternal and offspring health both in short- and long-term [2], the latter including increased risk for maternal type 2 diabetes mellitus (T2DM) and cardiovascular disease (CVD) [3]. Short-term consequences include increased risk for preeclampsia, which in itself is an established risk factor for CVD [4]. Women who have had GDM in their pregnancy have an increased risk of type 2 diabetes and cardiovascular disease later in life compared to those with normoglycemic pregnancies [3]. Through meta-analyses, the size of this risk association has been estimated to be a relative risk of almost 10 for T2DM and almost 2 for cardiovascular disease [5–7]. Although the relatively higher progression rate to T2DM in these women partly accounts for the CVD risk increase, meta-analyses have shown that GDM per se carries a residual risk [5, 7].

Epidemiological studies indicate that a healthy diet and increased physical activity can reduce the risk of developing T2DM [8, 9]. The period after a pregnancy with GDM has been referred to as window of opportunity for prophylactic interventions that can reduce the risk of T2DM and related comorbidities [10]; however, adherence to recommended postpartum screening for DM2 appears to be low [11, 12]. eHealth (electronic health) is defined as the use of information and communication technology for health. It is emerging as a tool with the potential of transforming facets of our health care systems, including perinatal care, but their practical utility and advantages over standard care remains to be determined [13, 14]. Our aim was to systematically review the existing literature on follow-up regarding cardiovascular disease after gestational diabetes, the utility of eHealth technology for this purpose, and to identify research gaps.

## Methods

### Protocol and registration

Following the Joanna Briggs methodology [15], a review protocol was developed and published at Open Science Framework (osf.io/p5hw6) before initiating the literature search [16]. There were no major deviances from the published review protocol. We used the Preferred Reporting Items for Systematic reviews and Meta-Analyses extension for Scoping Reviews (PRISMA-ScR) Checklist; see Additional file 1.

### Literature search and eligibility criteria

A literature search was performed by one of the authors (BSF) with the help of a librarian at the University of Oslo Library in May 2022. Four databases were searched: Ovid MEDLINE, Embase, Maternity and Infant Care, and Cochrane Database of Systematic Reviews. Some database-specific adaptions were made to the search strategy for the different databases. Detailed information on the literature search is provided in Additional file 2. We included original research articles and systematic reviews with a population of nulli- and multiparous women with GDM in a previous pregnancy, where the concept involved follow-up regarding long-term cardiovascular risk after such a pregnancy, as well as the use of eHealth technologies as a tool in such follow-up. The context was health care settings in which women receive care after a GDM pregnancy from skilled health care workers. Additionally, we included guidelines from the International Federation of Gynecology and Obstetrics (FIGO) as well as national guidelines from the UK, Canada, Australia/New Zealand, Sweden, Denmark, and Norway. These guidelines were chosen due to having a comparable population and system of ante- and postnatal care as the Norwegian health care system, a rationale that is consistent with other reviews [17]. We limited the search to publications in languages mastered to fluency by the review authors (English, German, Norwegian, Swedish, or Danish), without any date of publication restriction.

### Screening, data charting process, and synthesis of results

The results were downloaded to the EndNote reference management software (version 20; Clarivate Analytics, USA) and transferred to Covidence, a web-based collaboration software platform that streamlines the production of systematic and other literature reviews (Covidence systematic review software, Veritas Health Innovation, Melbourne, Australia, available at www.covidence.org).

Titles and abstracts were reviewed by two of the authors (BSF and MS) for relevance. All articles deemed to be relevant or of uncertain relevance underwent full-text review. In both stages of the process, if consensus was not reached, a third reviewer (ASDP) also reviewed and cast the deciding vote. The reference lists of the selected publications were manually searched for additional relevant articles.

A data charting form was developed (by all the authors) and completed for each study by BSF and MS independently. Data retrieved included information such as country of origin, methods, population, intervention, and outcomes. Certain information was extracted for guidelines that was not extracted for the primary studies and reviews and vice versa. The data charting form is provided in Additional file 3. The final version of the data charting form was expanded compared to the original one published with the protocol, but this was done in accordance with the planned testing and alignment of the data charting form early in the process. As outlined in the protocol, prior to starting the data charting stage of the review, two of the researchers (BSF and MS) tested and validated the form by independently screening three articles, comparing the results and adjusted the form to incorporate relevant findings. The final version of the data charting form was uploaded to the Covidence software platform, where both researchers doing the data charting (BSF and MS) used this independent of each other. In cases where the software flagged discrepancies in the data charted, the two researchers assessed the conflict and reached a consensus. The final version of the data charting form for each article or review then provided the basis when one researcher (BSF) created the first draft of the different tables in summarizing the results, which were then assessed by all the authors. In accordance with the JBI framework, no formal quality assessment of the scientific articles was performed; inclusion depended solely on the eligibility criteria.

## Results

**Fig. 1:**
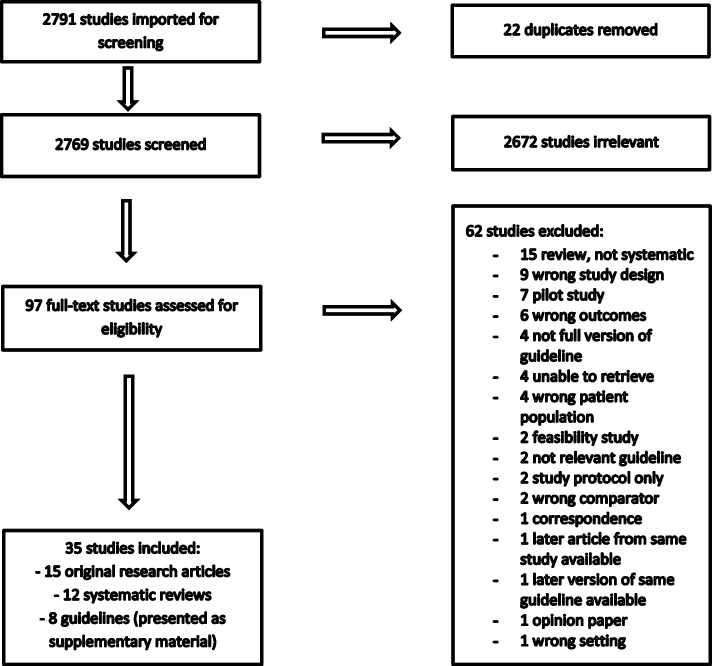
PRISMA flowchart

The search of the databases identified 2772 references. A further nine studies and systematic reviews were added after manual search of the reference list of other included articles. Ten guidelines also needed to be imported manually to obtain the full version in the original language. We removed 22 duplicates. Hence, in total, 2769 articles were screened for titles and abstract. This process excluded a further 2672 due to lack of relevance to the topic of interest. In total, 97 papers then underwent full-text review, in which 62 were excluded. Finally, 15 articles and 12 systematic reviews were included in the review. Data from eight gestational diabetes guidelines are presented in Additional file 4. A PRISMA flowchart of the process is shown in Fig. 1. An overview of the various definitions of GDM used in the different studies can be found in Table 1.

**Table 1:** The criteria for GDM used in the various studies

1999 WHO criteria [18]:	Women with impaired glucose tolerance (2-h plasma glucose ≥ 7.8 mmol/l (140 mg/dl) and < 11.1 mmol/l (200 mg/dl)) or diabetes mellitus (2-h glucose ≥ 11.1 mmol/l (200 mg/dl)) both categorized as having GDM
2006 WHO-criteria [19]:	Fasting plasma glucose ≥ 7.0 mmol/l (126 mg/dl) or 2-h 75 g OGTT plasma glucose ≥ 11.1 mmol/l (200 mg/dl)
2013 WHO/IADPSG criteria [20, 21]:	Fasting glucose 5.1–6.9 mmol/l (92–124 mg/dl) or 1-h plasma glucose ≥ 10.0 mmol/l (180 mg/dl) or 2-h plasma glucose 8.5–11.0 mmol/l (153–198 mg/dl) following a 75-g OGTT
Carpenter and Coustan criteria [22]:	5.3 mmol/l (95 mg/dl), 10.0 mmol/l (180 mg/dl), 8.6 mmol/l (155 mg/dl), and 7.8 mmol/l (140 mg/dl) plasma glucose values for fasting, 1-h, 2-h, and 3-h respectively after 100 g OGTT
1998 Australasian Diabetes in Pregnancy Society criteria [23]:	Fasting plasma glucose ≥ 5.5 mmol/l (99 mg/dl), or 2-h glucose ≥ 8.0 mmol/l (144 mg/dl) on a 75-g OGTT, or a glucose challenge test result ≥ 11.1 mmol/l (200 mg/dl)

*Abbreviations*: *GDM* Gestational diabetes mellitus, *WHO* World Health Organization, *OGTT* oral glucose tolerance test, *IADPSG* The International Association of Diabetes and Pregnancy Study Groups

No studies or systematic reviews were identified assessing long-term CVD risk per se; rather, the outcomes assessed were either incidence of T2DM or other markers of impaired glucose homeostasis or various CVD risk factors such weight and physical activity.

### Trials

#### Follow-up studies regarding cardiovascular risk

Eleven trials assessing follow-up regarding cardiovascular risk (excluding those with a primarily eHealth technology-based intervention, see the “The use of eHealth technologies” section) were deemed to meet the criteria of the review [24–34], of which seven were RCTs [26–28, 30–33], two cluster RCTs [24, 25], one randomized clinical with two interventions and no control group [34], and one an interventional cohort trial [29]. Details on the different trials are shown in Table 2. In four of the included trials, the primary outcomes were partly or exclusively related to postpartum weight change [24, 25, 27, 28], in three studies incidence of T2DM [26, 30, 32], and three studies reported other measures of glycemic status [28, 29, 31, 33, 34]. Glucose-related outcomes were additionally included among the secondary outcomes in two of the articles [25, 27]. The interventions were all different types of lifestyle interventions, focusing on diet and/or physical activity, delivered as either individual or group sessions, with different tools utilized as part of the follow-up (e.g., reminder systems using telephone or e-mail). Follow-up varied between six and 36 months for all the studies except Aroda et al., where follow-up was 10 years [32]. These authors found that compared to the placebo/standard care group the intensive lifestyle intervention reduced progression to diabetes by 35%. It should be noted that the setting of this trial was somewhat differing from the other studies, given that mean time since index GDM pregnancy was 12 years at the time of recruitment, a longer interval than in any of the other studies. In a smaller study, involving 180 participants, with two years follow-up, Zilberman-Kravits and co-workers found that a culturally tailored lifestyle intervention significantly reduced insulin resistance [33]. None of the other studies with incidence of T2DM or other glucose homeostasis-related outcomes found a significant effect. Three of the studies found significant effect of intervention on weight outcome [24, 25, 28]. Neither was there any consistent, significant effect on blood pressure, lipid profiles, or development of metabolic syndrome [24, 26, 29–31]

**Table 2:** Findings from the follow-up studies regarding cardiovascular risk

Author Year published Country	Study design and GDM definition	Aim of study	Population	Intervention	Outcomes measured	Key findings
- Ferrara et al.[24] - 2016 - USA	- Cluster RCT 44 facilities - Carpenter and Coustan criteria	- Comparing effectiveness of diabetes prevention strategies regarding postpartum weight retention	- 2280 women with previous GDM - Int.: 1087. Con.: 1193 - 84% retention rate for the 12-month survey - In intervention group, 50.3% completed one or more telephone session, 15.3% all 13	- Diabetes Prevention Program derived lifestyle intervention: gestational weight gain recommendations sent by mail and 13 telephone sessions between 6 weeks and 6 months postpartum - Control group received standard care: mailed reminder of postpartum diabetes screening and general lifestyle advice - Follow-up: 12 months	- Primary: Proportion of women meeting postpartum weight goals (reaching pregravid weight if pregravid BMI < 25 or losing 5% of pregravid weight if BMI ≥ 25.0) - Secondary: postpartum changes in daily total energy intake, percentage of calories from fat, physical activity, hypertension and depression	- Odds ratio for meeting weight goals 1.28 [95% CI 1.10, 1.47]. Greater increase in vigorous physical activity in intervention group: mean difference 15.4 min/week [4.9, 25.8]. No significant differences for other secondary outcomes
- Holmes et al. [25] - 2018 - Northern Ireland	- Cluster RCT Two facilities - 2013 WHO/IADPSG- criteria	- Determining the effectiveness of a postpartum lifestyle intervention program for overweight women with previous GDM	- 60 overweight (BMI ≥ 25) women with previous GDM. Int.: 29. Con.: 31 mean BMI 34.1 ± 6.3 in intervention group, 33.6 ± 5.4 in control group - In total 15 patient withdrawals	- A 1-h educational session at 6 weeks postpartum, free voucher for 3-month membership in commercial weight management organization, pedometer and intermittent follow-up with health educator via telephone/text message - Control group received standard care alone: educational DVD provided routinely when diagnosed with GDM - Follow-up: 6 months	- Primary: weight loss at 6 months Secondary: blood glucose, waist circumference, BMI	- Significant difference in reduction of weight (− 4.5 kg [− 8.1, − 0.9]), BMI and waist circumference. No significant difference in glucose measurements
- McManus et al. [27] - 2018 - Canada	- RCT - Not explicitly defined	- Evaluate the impact a postpartum healthy living program for women with recent GDM	- 170 overweight (predicted postpartum BMI ≥ 25) women with history of GDM - Int.: 89. Con.: 81. - 63 partners, results presented separately - 57% of included women completed the 12-month follow-up	- One-on-one healthy living-seminar, invitation to 1-h weekly walking group. Access to informational web-site. Automatic e-mail - Control group were provided with a hand-out from the Canadian Diabetes Association - Follow-up: 12 months	- Primary: Percentage of women reaching postpartum weight goal (losing ≥ 7% of postpartum weight) - Secondary included HbA1c, waist circumference, lifestyle habits, program participation, and engagement	- No significant differences for neither primary outcome nor waist circumference, HbA1c, physical activity, or diet
- O’Reilly et al. [28] - 2016 - Australia	- RCT - Australasian Diabetes in Pregnancy Society criteria^a^	- Evaluate a diabetes prevention program tailored to the needs of young mothers with previous GDM in the first postpartum year	- 573 women with previous GDM - Int.: 284. Con.: 289 - Only 10% of women attended all sessions. Loss to follow-up 27% (int.) and 21% (con.)	- One individual session, five group sessions, two telephone sessions consisting of diabetes risk assessment, diet and physical activity advice, stress management, etc. - Control group: usual care (not specified) - Follow-up: 12 months	- Primary: fasting blood glucose, weight, and waist circumference at 12 months - Secondary: changes in lifestyle parameters, depression scores, and CVD risk factors	- A 1 kg weight difference between the groups at 12 months was the only significant finding
- Lee et al. [26] - 2022 - Malaysia	- RCT - 2013 World Health Organization criteria	- Evaluate a system-based, postpartum intervention for women with GDM, aiming to reduce the incidence of diabetes and improve postnatal metabolic profiles	- 298 women with previous GDM - Int.: 130. Con.: 168. - Loss to follow-up: 54.2%	- One session of individualized health education during pregnancy (GW 36), booklet on diabetes prevention, five educational sessions postpartum, including one session with counseling by dietician and physiotherapist 6 weeks postpartum - Control group: standard care (group therapy on diet and physical activity during pregnancy - Follow-up: 2 years	- Primary: progression to T2DM - Secondary: changes in glucose levels, blood pressure, weight, lipid profiles	- No significant difference in primary outcome - For secondary outcomes only significant for diastolic blood pressure and triglycerides
- Rautio et al. [29] - 2014 - Finland	- Interventional cohort study - Not explicitly defined for previous GDM	- Comparing CVD risk profile and effect of a 1-year lifestyle intervention program in at-risk women ≤ 45 years with and without previous GDM	- 265 women ≤ 45 years with high risk of T2DM (previous GDM, history of impaired fasting glucose, impaired glucose tolerance or coronary heart disease, who made a least one intervention visit - 115 previous GDM - 150 no previous GDM	- Group interventions (exercise/weight maintenance groups, lectures on diabetes and related topics) - Individual counseling on diet, physical activity, smoking, alcohol, etc. - Follow-up: 12 months	- Primary: glucose tolerance. - Secondary: other cardiovascular risk factors, such as weight/BMI, blood pressure, lipid profiles	- Women previous GDM were younger and had a better CVD risk profile at baseline than women with no GDM-history - Both groups saw improvements in certain CVD risk factors, but there were no significant differences between the 2 groups, except for LDL cholesterol (− 0.21 vs − 0.09, *p* = 0.014)
- Shek et al. [30] - 2014 - China	- RCT - 1999 WHO criteria	- Assess whether lifestyle intervention can reduce risk of T2DM and metabolic syndrome in women with previous GDM	- 450 women with previous GDM and impaired glucose tolerance postpartum - Int.: 225. Con.: 225 - 6% loss to follow-up	- Individual counseling by dietician and later research nurse on dietary advice and exercise - Control: no treatment - Both groups were followed up twice at 3-monthly interval, then every 6 months - Follow-up: 36 months	- Primary: incidence of T2DM - Secondary: development of metabolic syndrome	- Not significant difference between the 2 groups for the primary outcome - No consistent, significant difference for the different parameters of metabolic syndrome
- Tandon et al. [31] - 2022 - India, Sri Lanka and Bangladesh	- RCT - IADPSG criteria	- To examine whether a lifestyle intervention could prevent worsening glycemic status in South Asian women with recent GDM	- 1601 women with GDM within the previous 18 months - Int.: 800. Con.: 801. - 20.1% loss to follow-up	- Four 90-min group sessions with advice on diet and exercise. 2 individual sessions for overweight participants. Sessions were adapted to local context and resources - Control: standard care - Follow-up: 12 months	- Primary: deterioration of glycemic status - Secondary: blood pressure, weight, waist circumference, etc.	- No statistically significant changes between the 2 groups for neither primary nor secondary outcomes
- Aroda et al. [32] - 2015 - USA	- RCT - Self-reported history of GDM	- Examine the effect of intensive lifestyle intervention and Metformin over 10 years on the risk of developing T2DM in women with and without a history of GDM	- 350 women with a history of GDM and 1416 parous women with no GDM history, with impaired glucose homeostasis and elevated BMI at time of enrolment - Mean time since index GDM pregnancy 12 years	- Intensive lifestyle intervention: 16-lesson core curriculum on diet, exercise and behavior modification aiming to achieve ≥ 7% weight loss and ≥ 150 min of moderate intensity physical activity per week - Later individual and group sessions to maintain any behavioral changes - Metformin group: 850 mg 1–2 times daily - Participants in placebo and metformin group received standard, non-intensive lifestyle advice - Follow-up: 10 years	- Incidence of T2DM	- In women with previous GDM, intensive lifestyle intervention reduced progression to diabetes compared to placebo by 35%. The corresponding figure for Metformin was 40%
- Zilberman-Kravits et al. [33] - 2018 - Israel	- RCT - Not specified	- Examine the efficacy of a culturally tailored lifestyle intervention on risk profile for T2DM after GDM	- 180 women with previous GDM. - Int.: 103, con.: 77 - Jewish and Bedouin ethnicity - 39% loss to follow-up after 2 years	- Culturally tailored lifestyle intervention with three counseling sessions with a nurse, and then 2-4 group meetings with advice on physical activity, diet, etc. - Control group received information on the increased risk of subsequent GDM and overt diabetes associated with GDM but no additional counseling sessions - Follow-up: 2 years	- Primary: homeostasis model assessment insulin resistance (HOMA-IR) - Secondary: lipid profile	- Significantly reduced HOMA-IR-levels in intervention group and improved lipid profiles
- Shyam et al. [34] - 2013 - Malaysia	- Randomized clinical trial. 2 different interventions, no control group - 2006 World Health Organization criteria	- Examining the effect of conventional dietary recommendations with and without the addition of low-GI education on glucose tolerance and body weight after GDM	- 77 women with previous GDM and one or more additional T2DM risk factors (BMI > 23, waist circumference > 80 cm, impaired glucose tolerance or impaired fasting glucose or a family history of T2DM) - 38 in conventional healthy dietary recommendations (CHDR) group - 39 in CHDR+Low-GI group - 19.4% withdrawal/drop-out	- Two diets with similar energy (max 1800 kcal/day) and macronutrient content but with different glycemic index - CHDR group received conventional dietary advice (low fat/refined sugar, high fiber, energy restriction). The CHDR+low-GI group received this advice but were additionally recommended to substitute high-GI foods with low-GI alternatives - Individual session with nutritionist at baseline, later two electronic interactions (either SMS or e-mail) per month - Participants were also recommended moderate physical activity for 30 m at least five times per week - Follow-up: 6 months	- Primary: 2 h 75 g OGTT glucose level - Secondary: fasting blood glucose, fasting serum insulin, weight/BMI, waist/hip circumference	- For the primary outcome, there was no significant reduction in 2h OGTT glucose level in the LGI group (− 0.2 mmol/l, *p* = 0.960), but the CHDR group saw a significant increase (0.8 mmol/l, *p* = 0.01), making the group difference statistically significant (*p* = 0.025) - For the secondary outcomes, there was a significant difference favoring LGI in BMI change and percentage of participants reaching weight goal

*Abbreviations*: *OGTT* Oral glucose tolerance test, *HbA1c* Glycated hemoglobin, *CVD* Cardiovascular disease, *T2DM* Type 2 diabetes mellitus, *GDM* Gestational diabetes mellitus, *BMI* Body mass index, *RCT* Randomized controlled trial, *Int*. Intervention group, *Con*. Control group, *GW* Gestational week, *WHO* World Health Organization, *IADPSG* The International Association of Diabetes and Pregnancy Study Groups

^a^2014 version is referenced in article, but the authors write that the definition was the criteria “at the time of study commencement,” with the cut-offs given in the text taken from 1998 version, which is the one that is cited in the study protocol

#### The use of eHealth technologies

Four studies on the use of eHealth measures met the criteria for this review [35–38], three utilizing smartphones [35, 37, 38] and one with the additional use of a virtual reality (VR) headset [37]. The fourth study tested the efficacy of a pedometer program linked with a web-based module, in addition to a nutrition coaching workshop [36]. Detailed information can be found in Table 3. The two trials with strictly smartphone-based interventions [35, 38] did not show significant effect on their primary outcomes of weight [38] and proportion of participants achieving a certain level of Diabetes Prevention Program goals [35] or secondary outcomes related to glucose levels or lipid profiles. Both applications were found to be acceptable by participants, as assessed by data on actual use of the apps [35, 38] and a scoring system where the users rated the app’s quality and perceived impact [35] Examining the efficacy of a mobile VR program [37], Kim and co-workers in a study from South Korea found that it significantly improved body weight and fat, fasting blood glucose, and HbA1c compared to control group after a 12-week follow-up. This was a quasi-experimental study, where 64 women with recent diabetes were included, and the control group of 64 women were selected to the intervention group by matching for age, birth experience, type of birth, family history of T2DM, and breastfeeding status. In a small trial with 31 women and 3 months follow-up, Peacock et al. [36] demonstrated a significant difference in weight loss in pedometer program intervention group compared to the control group (− 2.5 kg (SD ± 1.4) vs 0.0 kg (SD ± 2.3), *p* = 0.002).

**Table 3:** Findings from the eHealth studies

Author Year published Country	Study design and GDM definition	Aim of study	Population	Intervention	Outcomes measured	Key findings
- Kim et al. [37] - 2021 - South Korea	- Quasi-experimental study - Exact diagnostic not specified, only stated that it is based on the result of oral glucose tolerance test between gestational weeks 24 and 28	- Developing and evaluating the efficacy of a self-management mobile virtual reality program for preventing T2DM in women with recent GDM	- 128 women with recent GDM and no visual, auditory or active disabilities - Int.: 64. Con.: 64 - Control group not randomly assigned but selected after completing baseline measures for intervention group by matching for age, birth experience, type of birth, family history of T2DM, and breastfeeding status -5.4% patient withdrawal	- Mobile virtual reality program with diet and exercise modules, laughter therapy, and neonatal first aid - Control: written educational material - Follow-up: 12 weeks	- Body weight, body fat, fasting glucose, and HbA1c - Diabetes knowledge score - Dietary habits - Health promoting lifestyle score - Parenting stress	- Significant differences between the groups for all four physiological variables, as well as dietary habits and health promoting lifestyle score - No statistically significant differences for diabetes knowledge or parental stress
- Lim et al. [38] - 2021 - Singapore	- RCT - 2013 WHO/IADPSG criteria	- Examine the effectiveness of a smartphone app for restoring target weight postpartum in women with recent GDM	- 200 women with recent GDM - Int.: 101.Con.: 99 - 9% lost to follow-up	- Smartphone app where participants could log weight, meals, activity and interact with health care professionals - Control group: standard care, with dietary advice and OGTT 6 weeks postpartum and further follow-up based on results - Follow-up: 4 months	- Primary: attainment of target weight 4 months postpartum (return to first trimester weight if BMI ≤ 23, or a minimum of 5% weight loss from first trimester weight if BMI ≥ 23 - Secondary: HbA1c, lipid profiles and other biomarkers, blood pressure, absolute weight loss, self-report calorie intake, health behavior, and emotional distress scores	- No significant group differences for primary outcome nor for serum biomarkers - Self-reported calorie intake lower in intervention group, and higher health behavior score, but also higher level of emotional stress
- Potzel et al. [35] - 2022 - Germany	- RCT - Validated diagnosis of GDM according to German guidelines from 2014 (equivalent to 2013 WHO criteria)	- To evaluate the acceptability and effectiveness in improving CVD risk factors in women with recent GDM	- 66 women with GDM in last 3–18 months - Int.: 33. Con.: 33. - 18.2% drop-out	- iPhone app with modules on mental and emotional habits, physical activity, nutrition, and sleep. Possibilities for interaction with health care providers. Control: standard care (leaflet with lifestyle advice for preventing diabetes) - Follow-up: 6 months	- Primary: proportion of participants achieving ≥ 3 out of 5 Diabetes Prevention Program goals regarding physical activity, fiber and fat intake, and weight reduction/maintenance - Secondary: glucose levels, insulin sensitivity, oxygen uptake, body fat, and psychosocial indices. Patients also rated app acceptability	- No significant group differences for primary or secondary outcomes - 22 vs 11% in intervention and control group achieving primary outcome, but not statistically significant (*p* = 0.20) - App was well accepted
- Peacock et al. [36] - 2015 - Australia	- RCT - Not specified	- Developing a program to support behavior changes in order to delay or prevent T2DM in women with a history of GDM and BMI > 25	- 31 women with a GDM diagnosis within the previous 6 to 24 months - Int.: 16. Con.: 15 - 31% and 20% drop-out in intervention and control group respectively	- Pedometer program linked to web-based program where steps were logged and weekly goals generated. Also, 4 weeks nutrition coaching workshop - Control: “wait-list group,” offered nutrition workshop after end of follow-up Follow-up: 3 months	- Primary: weight loss - Secondary: waist/hip circumference, diet quality, insulin sensitivity, body composition, physical activity, self-efficacy in eating behaviors	- Significant difference in weight loss: − 2.5 kg (1.4 SD) vs 0.0 kg (2.3 SD), *p* = 0.002 - For the secondary outcomes, there were significantly better results in intervention group for hip and waist circumference and self-efficacy in eating behaviors

*Abbreviations*: *HbA1c* Glycated hemoglobin; *T2DM* Type 2 diabetes mellitus; *GDM* Gestational diabetes mellitus; *BMI* Body mass index, *RCT* Randomized controlled trial, *Int*. Intervention group, *Con*. Control group

### Reviews

Twelve reviews in total were included [39–50], of which one scoping review [39], one overview of other reviews [44], five systematic reviews [42, 45, 46, 48, 49], and five systematic reviews with meta-analyses [40, 41, 43, 47, 50]. Two of the reviews focused primarily on mHealth (mobile Health)/eHealth [39, 43], while the others mainly assessed lifestyle interventions. Of the two mHealth/eHealth reviews, the scoping review merely presented the existing literature and noted good engagement for app usage, but also a lack of studies where mHealth was the primary mode of intervention postpartum [39]. In their systematic review and meta-analysis, Halligan et al. [43] found that the results of the meta-analysis favored intervention compared to standard care for the outcomes of weight and BMI, but the results were not statistically significant. The meta-analyses of the lifestyle interventions showed somewhat mixed results. Li et al. [47] found that lifestyle interventions commenced within 3 years postpartum showed a 43% risk reduction for incidence of T2DM compared to standard care (RR 0.57, 95% CI 0.42–0.78), whereas the other reviews examining this outcome found statistically non-significant trend towards risk reduction [41, 50] or no effect for glucose related outcomes [40]. Hedeager Momsen and at al. [44] found in their overview of the reviews that lifestyle interventions appeared to decrease the incidence of diabetes postpartum and that the effects were larger the earlier after labor the intervention was implemented and the longer it lasted. The two meta-analyses for weight-related outcomes both showed small but statistically significant effects [40, 41]. In a review by Jones et al. [46], recruitment rates of participants to the various trials were assessed and found to be low even for primarily home-based interventions. In the systematic reviews overall, there were mixed results, but with most concluding that for outcomes such as weight/BMI, physical activity, and diet, lifestyle interventions may be beneficial. Details on the reviews is shown in Table 4.

**Table 4:** Findings from the reviews

Author Year Country	Types and number of included studies		Methods/participants	Aim of review	Outcomes assessed in review	Description of interventions	Results
- Edwards et al. [39] - 2022 - UK	- 30 articles included overall in review - 6 in a postpartum setting		- Scoping review - 6 databases searched: MEDLINE, CINAHL, Embase, Cochrane Library, Scopus, and TRIP - Women with known risk of GDM, currently having GDM or having had GDM previously - For the purpose of the present review, only the section focusing on postpartum studies was considered	- Examine the literature on mHealth as primary mode of preventing T2DM after GDM	- Efficacy not assessed, merely presenting the characteristics, content and implementation of the various mHealth technologies in the different studies, and whether these incorporated behavior change theory/techniques	- mHealth interventions, e.g., smartphone apps, wearable sensors, social media. Where mHealth was part of a broader intervention, it had to be considered the primary mode of intervention to be included in the review	- For the postpartum studies, no assessment of the efficacy ameliorating cardiovascular risk was presented in the review - Good engagement noted for two apps - The authors note a lack of studies were mHealth was the primary mode of intervention postpartum
- Gilinsky et al. [40] - 2015 - UK	- 13 studies included in systematic review - 5 in meta-analysis for weight outcomes - 4 included in meta-analysis for fasting blood glucose outcome		- Systematic review and meta-analysis - 4 databases searched: Web of Science, CCRCT, Embase, and Science DIRECT - Women with a previous GDM diagnosis	- Systematically review lifestyle interventions for women with previous GDM and assess changes in diet, physical activity, anthropometric outcomes and glycemic control and diabetes risk	- For systematic review: changes in activity, diet, and anthropometric outcomes For meta-analysis: weight loss, glycemic control, and T2DM risk	- Physical activity: 3 - Diet: 2 - Combination of diet and physical activity: 8	- 6 of 11 studies with a physical activity intervention reported effect - All 6 studies on diet interventions reported a favorable effect - Meta-analysis showed significant weight loss effect: Weighted mean difference= -1.06 kg (95% CI = − 1.68, − 0.44, *p* < 0.0.1),, but this was attributable to the effect reported in one Chinese study [51] that was excluded from this review on the grounds of being a feasibility study/interim report - Not significant results for glycemic control or T2DM risk
- Goveia et al. [41] - 2018 - Brazil	- 15 RCTs included in systematic review - 14 included in the various meta-analyses: 8 for T2DM, 9 for fasting glucose, 7 for 2h glucose level, 10 for BMI, 9 for weight, and 7 for waist circumference		- Systematic review and meta-analysis - 5 databases searched: PubMed, Cochrane Central Register of Controlled Trials, Web of Science, and Embase -Women with a previous GDM diagnosis	- Systematically review RCTs with lifestyle interventions in a postpartum setting for women with previous GDM	- Primary: incidence of T2DM or changes in glycemia - Secondary: changes in weight or waist circumference	- Lifestyle interventions - 10 studies tested a combination of diet and exercise - 1 study diet, exercise, and breastfeeding - 3 studies exercise alone - 1 study diet alone	- 8 studies assessing incidence of T2DM showed a borderline significant risk reduction of 25% (RR = 0.75, 95% CI 0.55–1.03). No significant effect for the other glycemic measures. Small, but statistically significant reductions for weight (MD = –1.07 kg; 1.43–0.72), BMI (MD = –0.94; –1.79 –0.09), and waist circumference (MD = − 0.98 cm; − 1.75–0.21)
- Guo et al. [42] - 2016 - China/USA	- 12 RCTs included in systematic review		- Systematic review. - 11 databases searched: PubMed, Embase, CINAHL, Cochrane Reviews, CENTRAL, Scopus, PsycINFO, ERIC, Wan Fang Data, China Knowledge Resource Integrated Database, and VIP Data - Women with previous GDM	- Assess outcomes of RCTs concerning diabetes prevention with previous GDM	- Incidence of T2DM, insulin resistance and weight-related outcomes	- 6 studies a combination of diet and physical activity, (with or without psychosocial support measures) - 1 diet, physical activity, and breastfeeding - 3 exercise (with or without psychosocial support) - 2 diet (with or without psychosocial support)	- 10 studies reported on T2DM development; of these, 7 studies reported at least a small, positive effect - 5 studies with at least a small effect size on insulin resistance; for weight-related measures, the corresponding figure was 11 of the 12 studies
- Halligan et al. [43] - 2021 - UK	- 14 RCTs in systematic review, 6 included in meta-analyses	- Systematic review and meta-analysis - Five databases searched: Embase, MEDLINE, CINAHL, PsycINFO, and The Cochrane Library - Women with previous GDM		- Comparing the effectiveness of digital/telemedicine interventions with standard care	- Primary: weight and BMI for meta-analyses - Secondary: waist circumference, fasting plasma glucose, physical activity, and diet	- 6 studies where telephone contact was primary intervention - 4 webprogram/-platform - 1 smartphone app - 3 SMS	- For both weight (− 1.83 kg, 95% CI − 4.08–0.42, *p* = 0.11) and BMI (− 0.9, 95% CI − 1.89–0.09, *p* = 0.08), the meta-analysis favored intervention, but not statistically significant - For the secondary outcomes of the systematic review, there were no clear, statistically significant patterns
- Hedeager Momsen et al. [44] - 2021 - Denmark	- 18 systematic reviews included	- Overview of reviews, using the principles from the Joanna Briggs Institute (JBI) methodology - Six databases searched: Cochrane Library, PubMed, JBI, Embase, CINAHL, Web of Science - Women with previous GDM		- Presenting an overview of reviews for T2DM prevention interventions in women after GDM, in order to use this information for establishing local interventions	- Incidence of T2DM, encouragement of healthy behavioral changes and consequences for the woman’s own perception of increased T2DM risk	- 7 reviews primarily lifestyle (diet and/or physical activity) interventions - 6 reviews with both lifestyle and pharmacological interventions - 6 reviews also included breastfeeding promoting interventions - 1 review examining determinants for adherence to physical activity, as well as its effectiveness - 3 reviews on screening regimes	- Lifestyle, breastfeeding and pharmacological interventions all appeared to decrease the incidence of postpartum T2DM - Effects larger the earlier after labor it was implemented, and the longer it lasted
- Huang et al. [45] - 2022 - USA	- 7 controlled intervention studies, of which 6 RCTs and one with a quasi-experimental design		- Systematic review - 7 databases searched: MEDLINE, CINAHL, Embase, Cochrane Central, Web of Science, PsycINFO, and ProQuest Dissertations and Theses - Women with current or previous GDM	- Investigate the characteristics and effectiveness of lifestyle interventions both in pregnancy and postpartum for preventing T2DM after GDM	- Glucose regulation, weight, lifestyle behaviors, and knowledge	- The postpartum interventions were all lifestyle interventions (diet and/or physical activity) - Varying modes of delivery of interventions: individual in-person visits, telephone, group sessions, SMS, and multimodular	- Glucose regulation: 1 study showing significant effect, 3 without effect - Weight change: 3 with effect, 3 without effect - Physical activity: 3 with effect, 4 no effect - Diet: 2 with effect, 3 no effect
- Jones et al. [46] - 2017 - USA	- 10 RCTs		- Systematic review - 2 databases searched: MEDLINE and CINAHL - Women with previous GDM	- Synthesize knowledge and practices regarding postpartum, multimodular lifestyle interventions to reduce risk of T2DM	- Weight loss, diet, physical activity - Recruitment rates	- Multimodal lifestyle interventions -Telephone and mail primary modes of contact in 7 studies - Website primary mode of contact in 3 studies	- Individualized interventions may improve outcomes regarding postpartum weight loss and dietary quality - Not significant for physical activity - Low recruitment rates even for primarily home-based interventions
- Li et al. [47] - 2021 - China	- 15 RCTs, 4 with intervention during pregnancy, 11 with intervention postpartum included in systematic review and meta-analysis		- Systematic review and meta-analysis - 17 databases searched: PubMed, Web of Science, Cochrane Library, Embase, SpringerLink, Wiley Online Library, Science Direct, MEDLINE, JAMA, CNKI, Wanfang Med Online, Lancet, Nature, Science, NEMJ, and Google Scholar - Women with GDM in index pregnancy, where data on postpartum diabetes was available	- Assess the effects of lifestyle interventions during and after pregnancy to reduce the risk of postpartum T2DM after a GDM pregnancy	- Risk of postpartum T2DM	- Lifestyle interventions (diet and/or physical activity)	- Lifestyle interventions commenced within 3 years postpartum showed a 43% risk reduction compared to standard care: RR 0.57, 95% CI 0.42–0.78)
- Morton et al. [48] - 2014 - UK	- 11 studies included in systematic review - 6 RCTs, 4 comparative cohort studies, one open-label observational study		- Systematic review - 5 databases searched: MEDLINE, Embase, CINAHL, Maternity and Infant Care, Cochrane Library - Women with previous GDM	- Examine the effectiveness of interventions for delaying or preventing T2DM after a GDM pregnancy	- Effect on T2DM risk factors and incidence of T2DM	- 6 studies (3 RCTs and 3 observational cohort studies): lifestyle interventions (diet and/or physical activity) - 4 studies (3 RCTs and 1 observational study): pharmacological (1 RCT where it was combined with lifestyle intervention) - 2 breastfeeding	- Trials involving lifestyle interventions indicated that they might be beneficial, but there was no consistent, statistically significant effect across the trials
- Pedersen et al. [50] - 2017 - Denmark	- 10 RCTs included in systematic review - 4 trials in meta-analysis		- Systematic review and meta-analysis - Four databases searched: PubMed, Cochrane Library, Embase, CINAHL - Women with previous GDM	- Examine the effectiveness of behavioral interventions on preventing T2DM after GDM	- 5 incidence of T2DM - 6 effect on biomarkers of insulin resistance - 9 trials weight change - The 4 trials included in meta-analysis had T2DM incidence as outcome	- 8 studies combination of diet and physical activity - 1 physical activity only - 1 diet only	- All the trials included in meta-analysis showed a tendency for reduction of incidence of T2DM, but not statistically significant. The pooled analysis showed a statistically significant risk difference in intervention group vs control of − 5.02 per 100 (95% CI: − 9.24 to − 0.80) - Four trials showed a statistically significant effect on weight/BMI, five trials no significant effect - The outcomes concerning markers of insulin resistance were inconsistent
- Peacock et al. [49] - 2014 - Australia	- 30 studies included in systematic review		- Systematic review - Three databases searched: PubMed, CINAHL, and MEDLINE	- Examine strategies and programs aimed at reducing risk of T2DM after GDM and the barriers to participation as well as the role of midwives in such programs	- Incidence of T2DM, dietary behavior change, weight loss, physical activity	- Mainly lifestyle interventions (diet, activity, etc.). 1 with a pedometer and web-based educational module. 3 of the RCTs and 1 observational also included pharmacological intervention, of which one RCT and one observational looking exclusively at the effect of a pharmacological intervention	- 5 of the 8 RCTs and 3 of 5 observational studies reported positive results for the intervention concerning T2DM incidence or related risk factors

*Abbreviations*: *HbA1c* Glycated hemoglobin, *T2DM* Type 2 diabetes mellitus, *GDM* Gestational diabetes mellitus, *BMI* Body mass index, *RCT* Randomized controlled trial

## Discussion

### Summary of evidence

The studies assessed in the present scoping review do not offer any clear evidence for how best to follow-up women after gestational diabetes regarding their increased long-term risk of cardiovascular disease. Various lifestyle interventions have been tested for outcomes such as diabetes incidence, weight-related outcomes, and other cardiovascular risk factors, most offering some version of patient education combined with individual or group sessions with health care professionals. The results from both primary studies and reviews indicate that such follow-up *may* be beneficial but differ between the various studies and reviews for the different outcomes to such a degree that it is not possible to conclude that any of them provide a clear template for how follow-up should be carried out.

The use of eHealth is increasing in health care systems across the world. In a recent WHO guideline [13] regarding the implementation of such measures, a degree of caution was advised, emphasizing the importance of rigorously evaluating their utility, to ensure that they do not divert resources from non-digital interventions if they are not superior. The eHealth interventions assessed in this review have not shown any clear and consistent advantages compared to standard care. However, it is possible that the lack of statistically significant results in the smartphone app trials [35, 38] was at least partly related to a relatively short follow-up period of 4 and 6 months, respectively. On the other hand, Kim et al. [37] found a statistically significant result after only a 12-week follow-up, but given the quasi-experimental design, a degree of caution is necessary when interpreting the results.

Another obstacle that needs to be overcome to improve the follow-up of this group of women after GDM is the low rate of adherence to existing follow-up recommendations such as postpartum glucose tolerance tests, which is attended by less than one in five [52]. Use of proactive reminder systems and mobile health technology have been suggested as possible remedies for this, and the latter highlighted as an area that warrants further research [14, 53].

Although it is established that women with a GDM pregnancy have a significantly increased risk of CVD later in life, even when adjusting for the risk of T2DM [5, 7], no studies were identified that assessed strategies for reducing overall cardiovascular risk. All the studies were focusing on either persistent hyperglycemia or other individual risk factors of CVD such as weight, diet, and physical activity. Taking into account that the risk of CVD to a certain degree is independent of the considerably increased incidence of T2DM in women with a previous GDM pregnancy compared to those without GDM, this is an area that warrants further research.

Given that women on average are relatively young and healthy at the time of reproduction, it might be that the next contact for a woman with prior GDM and a normal HbA1c or OGTT postpartum—if tested—might be the first trimester of a next pregnancy or even years later if she does not have any more children. This makes the pregnancy and peripartum period a missed window of opportunity for optimizing any modifiable cardiovascular risk factor. As presented in Additional file 4, guidelines generally suggest some type of postnatal testing for persistent hyperglycemia; however, few explicitly address the risk of CVD apart from T2DM, and the Norwegian guideline stands alone in offering a clear template for how this could be followed up. The Norwegian guidelines link obstetric outcomes with a follow-up by general practitioners in the public health system, which may assist in bridging the gap in the health follow-up of postpartum women. In patients with previous hypertensive disorders of pregnancy, guidelines such as NICE [54] and ACOG [55] recommend that women are followed by their primary care provider to manage risk factors for cardiovascular disease. The Norwegian guidelines [56] for hypertensive disorders of pregnancy offer a clear algorithm suggesting how this could be carried out from delivery to middle age and beyond, a flow-chart which has since been included also in the guidelines for gestational diabetes [57]. As both spectrums of obstetric disease confer an increased risk of CVD later in life, similar recommendations for follow-up might be a sensible approach. As the development of CVD is an insidious process developing over years [58], studies with a longer follow-up would be a welcome addition to the literature.

### Strengths and limitations

To the best of our knowledge, this is the most comprehensive review that has been performed for this topic, encompassing both original research studies and systematic reviews and including both more conventional lifestyle interventions and also with a separate assessment of the utility of eHealth technologies The scoping review methodology does not entail quality assessment; hence, our review has not analyzed the quality of the included studies. Our review was not able to generate evidence that supports any specific follow-up regime to lower the cardiovascular risk in women with previous gestational diabetes, whether using conventional methods or eHealth measures. Some limitations should be noted. First of all, the review protocol was not peer reviewed. Another limitation to our study was that the search did not yield the full, original language version of any of the included guidelines; hence, these had to be inserted manually. We also acknowledge that more databases could have been searched, including those cataloging grey literature. Another limitation is the language restrictions. In itself, the inability to review articles written in other languages than the ones the authors of this paper are fluent in could be a source of bias. In retrospect, we also acknowledge that it would be preferable to have included language among the eligibility criteria rather than as restrictions in the search strategy.

## Conclusions

Our scoping review has shown that although GDM is an established risk factor for CVD later in life, it is not possible to ascertain from the existing literature how women with a history of gestational diabetes mellitus should be followed up in this regard. The studies and reviews assessed in this scoping review suggest that lifestyle interventions may be beneficial for certain individual risk factors, such as weight-related outcomes and risk of T2DM, but no studies assessing the overall long-term risk has been performed. eHealth technology is not an established feature in the follow-up of women after GDM, and although such measures appear to be acceptable to participants, they have yet to prove their utility for improving follow-up and lowering cardiovascular risk.

There is need for further research on how best to follow-up concerning long-term risk of overall CVD after a GDM pregnancy. Studies with longer follow-up assessing how to utilize eHealth technologies would also be a welcome addition to the literature. The increased risk of CVD and parallel recommendations for hypertensive disorders of pregnancy suggest that similar approach regarding follow-up for optimizing cardiovascular risk factors could be reasonable. However, the existing literature does not offer any clear advice on how this should be carried out.

### Supplementary Information

**Additional file 1.** PRISMA-ScR Checklist.

**Additional file 2.** Documentation of literature search.

**Additional file 3.** Data charting form.

**Additional file 4.** Findings from the guidelines.

## Data Availability

Not applicable.
