# Correction: Gestational diabetes mellitus, follow-up of future maternal risk of cardiovascular disease and the use of eHealth technologies – a scoping review

**DOI:** 10.1186/s13643-023-02383-2

**Published:** 2023-11-16

**Authors:** Bendik S. Fiskå, Aase Serine Devold Pay, Anne Cathrine Staff, Meryam Sugulle

**Affiliations:** 1 Division of Obstetrics and Gynaecology, Oslo University Hospital, Oslo, Norway; 2 Institute for Clinical Medicine, Faculty of Medicine, University of Oslo, Oslo, Norway; 3 Faculty of Health Sciences, Oslo Metropolitan University, Oslo, Norway; 4 Department of Obstetrics and Gynaecology, Bærum Hospital, Vestre Viken Hospital Trust, Bærum, Norway

**Correction: Systematic Reviews 12, 178 (2023)**

10.1186/s13643-023-02343-w

Following publication of the original article [1], the authors found one mistake while reading the article. In Fig. 1 as follows, in the second box in the right column, it should be “2672 studies irrelevant”.

**Fig. 1:**
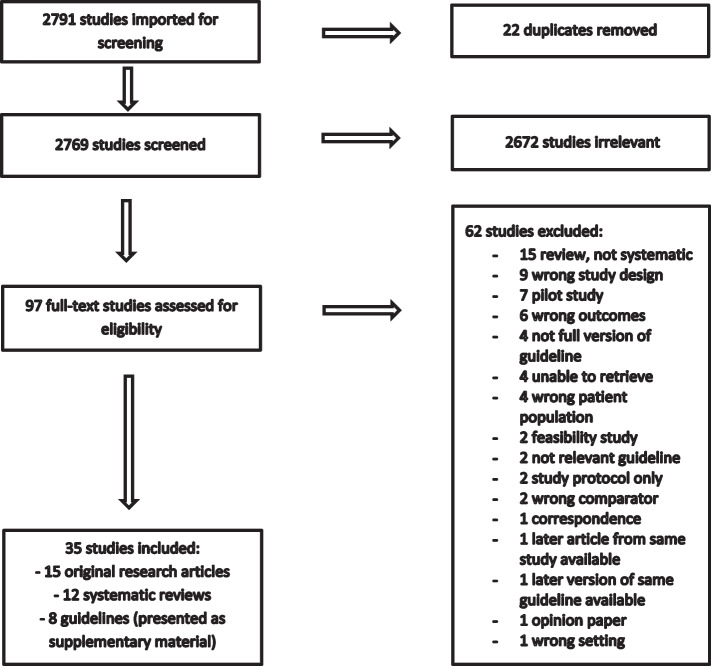
PRISMA flowchart

The correct figure is shown here and the original article has been corrected.
