# Supplementary material for: Gestational diabetes mellitus, follow-up of future maternal risk of cardiovascular disease and the use of eHealth technologies—a scoping review
Source: Syst Rev. 2023 Sep 28;12:178. doi: 10.1186/s13643-023-02343-w (PMC10537141; doi:10.1186/s13643-023-02343-w)
Supplement: Supplementary file 2 — Additional file 2. Documentation of literature search. [file 13643_2023_2343_MOESM2_ESM.pdf]

Documentation of literature search

Documentation on the literature search for:

Gestational diabetes mellitus: follow-up regarding long-term risk of cardiovascular disease and the use of eHealth technologies - protocol for a scoping review

The following databases were searched:

Database	Number of retrieved references
Ovid Medline	2555
Embase	1511
Maternity & Infant Care (MIDIRS)	271
Cochrane Database of Systematic Reviews	38
Number of references before deduplication:	4375
Number of references after deduplication:	2772

All searches were done 16 May 2022 by Marie Isachsen, librarian at the Medical library, University of Oslo.

Search syntax:

/	After an index term indicates a subject heading were selected
Exp	A subject heading are "exploded" automatically to retrieve citations that carry the specified subject heading as well as the more specific terms indented beneath it in the subject heading hierarchy. Example: The exploded heading “Telecommunications” in Ovid Medline will retrieve articles indexed with the subject headings Electronic Mail, Telemedicine, Remote Communication, Remote Sensing Technology, Smartphone etc.
.ti,ab,kf.	Search for a term in title, abstract and author keywords
*	At the end of a term indicates that this term has been truncated, diabet* retrieves diabetes, diabetic, diabetics
Adj	The Adjacent operator (ADJ) retrieves records with search terms next to each other in that specific order
Adj3	Search for two terms next to each other, in any order, up to 3 words in between.

Search strategies:

Ovid MEDLINE(R) ALL 1946 to May 11, 2022

#	Searches	Results
1	Diabetes, Gestational/	13683
2	(gestat* adj1 diabet*).ti,ab,kf.	18952

3	pregnancy-induced diabetes.ti,ab,kf.	18
4	gdm.ti,ab,kf.	9688
5	(pregnan* adj2 relat* adj2 diabetes).ti,ab,kf.	50
6	or/1-5	21545
7	Follow-Up Studies/ or "Continuity of Patient Care"/ or Aftercare/ or Postpartum Period/	742519
8	(follow-up* or followup* or aftercare or after-care or postpartum or post-partum or postnatal* or post-natal* or puerperium).ti,kf.	180549
9	((recent or previous or following or former or prior) adj (gestat* or pregnanc* or diabet*)).ti,kf.	860
10	((recently or previously) adj diagnosed adj2 diabet*).ti,kf.	62
11	(((recent or previous or following or former og prior) adj (gestat* or pregnanc* or diabet*)) and (follow-up* or followup*)).ab.	505
12	exp Computer Communication Networks/ or exp Telecommunications/ or exp Computing Methodologies/ or Computer-Assisted Instruction/ or Reminder Systems/ or exp Wearable Electronic Devices/ or Distance Counseling/	1232877
13	(internet or online or on-line or digital* or web or website* or weblog* or webcast or e-health or ehealth or electronic health or m-health or mhealth or mobile health or e-care or ecare or mobile care or m-care or mcare or e-medicine or e-therap* or etherap* or electronic based or e-based or mlearning or m-learning or mobile-learning or electronic learning or elearning or e-learning or electronic teaching or e-teaching or electronic education or e-education or ipad* or keypad* or tablet or tablets or laptop* or e-mail or email or electronic mail or chat or chatting or phone or phones or telephone* or iphone* or cellphone* or app or apps or smartapp* or smartphone* or smartwatch* or ((mobile or electronic or smart or sensor* or sensing) adj (technolog* or health technolog* or healthcare technolog* or application* or device* or watch*)) or (wearable adj (electronic or technolog*)) or android or text messaging or texting or sms or short message service* or mms or e-technolog* or multimedia or software* or podcast* or computer* or minicomputer* or telemedical or telemedicine or telecare or tele-care or telehome* or tele-home* or telehomecare or tele-homecare or telehomeconsultation* or teleconsultation* or tele-consultation* or telecounsel* or tele-counsel* or telerehabilitation or tele-rehabilitation or telemonitoring or tele-monitoring or telehealth or tele-health or telehealthcare or tele-healthcare or telecoach* or telecommunication* or telenursing or tele-nursing or information technolog* or reminder system* or wireless technolog* or video* or bluetooth or blue-tooth or social media or social medium or social networking site* or blog or blogs or blogging or weblog* or vlog* or microblog* or twitter or tweet* or tweeting or	435341

	facebook or Instagram or skype or skyping or zoom or youtube or ((remote or distance or distant) adj (rehabilitation or therap* or treatment or communicat* or consultat* or counsel* or sensing or sensor* or care or monitor* or technolog* or medical technolog*)) or (sensor* adj3 monitor*) or ((fitness or activit*) adj1 track*) or smarthome* or smarthous* or smartcare or (smart adj (home or house* or care or environment*)) or ambient assisted living or ambient intelligence or (virtual adj (realit* or environment*)) or exergam*).ti,kf.	
14	or/7-13	2257878
15	6 and 14	2664
16	(Animal Experimentation/ or exp Animals/) not Humans/	5006435
17	15 not 16	2583
18	limit 17 to (danish or english or german or norwegian or swedish)	2555

Embase Classic+Embase 1947 to 2022 May 11

#	Searches	Results
1	(gestat* adj1 diabet*).ti,ab,kf.	29981
2	pregnancy-induced diabetes.ti,ab,kf.	23
3	gdm.ti,ab,kf.	15277
4	(pregnan* adj2 relat* adj2 diabetes).ti,ab,kf.	88
5	or/1-4	31225
6	(follow-up* or followup* or aftercare or after-care or postpartum or post-partum or postnatal* or post-natal* or puerperium).ti,kf.	251647
7	((recent or previous or following or former or prior) adj (gestat* or pregnanc* or diabet*)).ti,kf.	1203
8	((recently or previously) adj diagnosed adj2 diabet*).ti,kf.	67
9	(((recent or previous or following or former or prior) adj (gestat* or pregnanc* or diabet*)) and (follow-up* or followup*)).ab.	1023
10	(internet or online or on-line or digital* or web or website* or weblog* or webcast or e-health or ehealth or electronic health or m-health or mhealth or mobile health or e-care or ecare or mobile care or m-care or mcare or e-medicine or e-therap* or etherap* or electronic based or e-based or mlearning or m-learning or mobile-learning or electronic learning or elearning or e-learning or electronic teaching or e-teaching or electronic education or e-education or ipad* or keypad* or tablet or tablets or laptop* or e-mail or email or electronic mail or chat or chatting or phone or phones or telephone* or iphone* or cellphone* or app or apps or smartapp* or smartphone* or	560498

	smartwatch* or ((mobile or electronic or smart or sensor* or sensing) adj (technolog* or health technolog* or healthcare technolog* or application* or device* or watch*)) or (wearable adj (electronic or technolog*)) or android or text messaging or texting or sms or short message service* or mms or e-technolog* or multimedia or software* or podcast* or computer* or minicomputer* or telemedical or telemedicine or telecare or tele-care or telehome* or tele-home* or telehomecare or tele-homecare or telehomeconsultation* or teleconsultation* or tele-consultation* or telecounsel* or tele-counsel* or telerehabilitation or tele-rehabilitation or telemonitoring or tele-monitoring or telehealth or tele-health or telehealthcare or tele-healthcare or telecoach* or telecommunication* or telenursing or tele-nursing or information technolog* or reminder system* or wireless technolog* or video* or bluetooth or blue-tooth or social media or social medium or social networking site* or blog or blogs or blogging or weblog* or vlog* or microblog* or twitter or tweet* or tweeting or facebook or Instagram or skype or skyping or zoom or youtube or ((remote or distance or distant) adj (rehabilitation or therap* or treatment or communicat* or consultat* or counsel* or sensing or sensor* or care or monitor* or technolog* or medical technolog*)) or (sensor* adj3 monitor*) or ((fitness or activit*) adj1 track*) or smarthome* or smarthous* or smartcare or (smart adj (home or house* or care or environment*)) or ambient assisted living or ambient intelligence or (virtual adj (realit* or environment*)) or exergam*).ti,kf.	
11	or/6-10	810128
12	5 and 11	2495
13	(exp animal/ or exp animal model/ or nonhuman/) not exp human/	7606023
14	12 not 13	2470
15	limit 14 to (danish or english or german or norwegian or swedish)	2384
16	limit 15 to conference abstracts	753
17	15 not 16	1631
18	limit 17 to (article or review)	1511

Maternity & Infant Care Database (MIDIRS) 1971 to April 26, 2022

#	Searches	Results
1	(gestat* adj1 diabet*).ti.	2903
2	pregnancy-induced diabetes.ti.	0
3	gdm.ti.	69
4	(pregnan* adj2 relat* adj2 diabetes).ti.	1

5	or/1-4	2948
6	(follow-up* or followup* or aftercare or after-care or postpartum or post-partum or postnatal* or post-natal* or puerperium).ti.	11931
7	((recent or previous or following or former or prior) adj (gestat* or pregnanc* or diabet*)).ti.	175
8	((recently or previously) adj diagnosed adj2 diabet*).ti.	0
9	(internet or online or on-line or digital* or web or website* or weblog* or webcast or e-health or ehealth or electronic health or m-health or mhealth or mobile health or e-care or ecare or mobile care or m-care or mcare or e-medicine or e-therap* or etherap* or electronic based or e-based or mlearning or m-learning or mobile-learning or electronic learning or elearning or e-learning or electronic teaching or e-teaching or electronic education or e-education or ipad* or keypad* or tablet or tablets or laptop* or e-mail or email or electronic mail or chat or chatting or phone or phones or telephone* or iphone* or cellphone* or app or apps or smartapp* or smartphone* or smartwatch* or ((mobile or electronic or smart or sensor* or sensing) adj (technolog* or health technolog* or healthcare technolog* or application* or device* or watch*)) or (wearable adj (electronic or technolog*)) or android or text messaging or texting or sms or short message service* or mms or e-technolog* or multimedia or software* or podcast* or computer* or minicomputer* or telemedical or telemedicine or telecare or tele-care or telehome* or tele-home* or telehomecare or tele-homecare or telehomeconsultation* or teleconsultation* or tele-consultation* or telecounsel* or tele-counsel* or telerehabilitation or tele-rehabilitation or telemonitoring or tele-monitoring or telehealth or tele-health or telehealthcare or tele-healthcare or telecoach* or telecommunication* or telenursing or tele-nursing or information technolog* or reminder system* or wireless technolog* or video* or bluetooth or blue-tooth or social media or social medium or social networking site* or blog or blogs or blogging or weblog* or vlog* or microblog* or twitter or tweet* or tweeting or facebook or Instagram or skype or skyping or zoom or youtube or ((remote or distance or distant) adj (rehabilitation or therap* or treatment or communicat* or consultat* or counsel* or sensing or sensor* or care or monitor* or technolog* or medical technolog*)) or (sensor* adj3 monitor*) or ((fitness or activit*) adj1 track*) or smarthome* or smarthous* or smartcare or (smart adj (home or house* or care or environment*)) or ambient assisted living or ambient intelligence or (virtual adj (realit* or environment*)) or exergam*).ti.	3140
10	or/6-9	15051
11	5 and 10	271

Cochrane Database of Systematic Reviews (searched via the "Ovid MEDLINE(R) ALL" interface)

#	Searches	Results
1	Diabetes, Gestational/	13683
2	(gestat* adj1 diabet*).ti,ab,kf.	18952
3	pregnancy-induced diabetes.ti,ab,kf.	18
4	gdm.ti,ab,kf.	9688
5	(pregnan* adj2 relat* adj2 diabetes).ti,ab,kf.	50
6	or/1-5	21545
7	Cochrane Database of Systematic Reviews.jn.	15812
8	6 and 7	55
9	Manually excluded systematic reviews with status "withdrawn", and older versions	38
