# Supplementary material for: Gestational diabetes mellitus, follow-up of future maternal risk of cardiovascular disease and the use of eHealth technologies—a scoping review
Source: Syst Rev. 2023 Sep 28;12:178. doi: 10.1186/s13643-023-02343-w (PMC10537141; doi:10.1186/s13643-023-02343-w)
Supplement: Supplementary file 3 — Additional file 3. Data charting form. [file 13643_2023_2343_MOESM3_ESM.pdf]

Data charting form

**Endnote-reference:**	
**Country of origin**	
**Name of society/organisation issuing guideline [Guidelines only]**	
**What were the recommendations based on? [Guidelines only].**<br>- Literature, systematic searches, consensus, other? Describe.	
**Study design/methods [primary studies/reviews]**	
**Type of study [primary studies], Types and number of included studies [reviews]**	
**Aim of study/review**	
**Definition of gestational diabetes [Guidelines and primary studies]**	
**Population (definition, number, characteristics) [Primary studies and reviews]**<br>- Characteristics: Age, BMI, risk factors, etc.	
**Intervention [primary studies]/description of interventions [reviews]**	
**Outcomes measured [primary studies/reviews]**	

**Results [primary studies/reviews]**	
**Recommendations for postpartum testing for diabetes [Guidelines only]**	
**Recommendations for follow-up regarding cardiovascular risk [Guidelines only]**	
**Other relevant information**	
