# Supplementary material for: Gestational diabetes mellitus, follow-up of future maternal risk of cardiovascular disease and the use of eHealth technologies—a scoping review
Source: Syst Rev. 2023 Sep 28;12:178. doi: 10.1186/s13643-023-02343-w (PMC10537141; doi:10.1186/s13643-023-02343-w)
Supplement: Supplementary file 4 — Additional file 4. Findings from the guidelines. [file 13643_2023_2343_MOESM4_ESM.docx]

Additional file 4– Findings from the guidelines

| Name of society/organization  Country  Year published | Basis for recommendations | Definition of GDM | Recommendations for postpartum testing for diabetes | Recommendations for follow-up regarding cardiovascular risk |
| --- | --- | --- | --- | --- |
| - The American College of Obstetricians and Gynecologists [1]  - USA  - 2018 | - Literature search, terms not provided.  - Other guidelines  - Expert opinion | - No single definition. Both Carpenter Coustan and National Diabetes Data Group criteria presented | - Screening (fasting blood glucose and/or 2-hour OGTT) recommended at 4-12 weeks postpartum  - Repeat testing for T2DM recommended every 1-3 years after GDM pregnancy where the postpartum screening was normal | - CVD risk not mentioned.  - Follow-up with primary care physician recommended, content of follow-up not specified |
| - Dansk Selskab for Obstetrik og Gynækologi (Danish Society of Obstetrics and Gynaecology) [2]  - Denmark  - 2010 | - Process not described. Four references given (two Danish consensus conferences and two other Danish guidelines) | - Blood glucose level ≥ 9.0 mmol/l after a 2-hour 75 g OGTT | - OGTT within 3-4 months postpartum  - OGTT or fasting blood glucose every 3 years | - CVD risk not addressed.  - Individual counselling on lifestyle, future diabetes risk and risk related to future pregnancies recommended |
| - Deutsche Diabetes Gesellschaft/Deutsche Gesellschaft für Gynäkologie und Geburtshilfe (German Diabetes Association/German Association for Gynaecology and Obstetrics) [3]  - Germany  - 2018 | - Scientific literature. Details on literature search not provided  - Graded level of strength of recommendations | - Based on 75g OGTT:  -Fasting blood glucose ≥ 92 mg/dl (5,1 mmol/l)  - 1-hour ≥ 180 mg/dl (10,0 mmol/l)  - 2-hour ≥ 153 mg/dl (8,5 mmol/l) | - Postpartum OGTT 6-12 weeks postpartum  - If this test shows abnormal fasting glucose or impaired glucose tolerance, annual measurement of fasting blood glucose and HbA1c is suggested | - CVD risk not mentioned  - Lifestyle advice recommended for lowering risk of T2DM |

Abbreviations: OGTT: Oral glucose tolerance test; HbA1c: glycated hemoglobin; CVD: Cardiovascular disease; T2DM: Type 2 diabetes mellitus; GDM: Gestational diabetes mellitus; WHO: World Health Organization; IADPSG: The International Association of Diabetes and Pregnancy Study Groups; GP: General practitioner

Additional file 4– Findings from the guidelines (continued)

| Name of society/organization  Country  Year published | Basis for recommendations | Definition of GDM | Recommendations for postpartum testing for diabetes | Recommendations for follow-up regarding cardiovascular risk |
| --- | --- | --- | --- | --- |
| - Canadian Diabetes Association[4]  - Canada  - 2018 | - Literature search. Terms not provided. Flow chart showing the selection/screening process and adherence to PRISMA guidelines | - “Preferred approach”: First a 50 g glucose challenge test, and if this is abnormal continue with a 75 g OGTT, where the diagnosis of GDM is made if one blood glucose value is abnormal (i.e. fasting ≥5.3 mmol/l, 1-hour ≥10.6 mmol/l, 2-hour ≥9.0 mmol/l). | - 75g OGTT 6 weeks to 6 months postpartum  - Reminder systems recommended to improve uptake of screening | - CVD risk later in life not addressed  - Counselling on healthy behaviors aiming to reduce risk of T2DM recommended |
| - Norsk gynekologisk forening (Norwegian Society for Gynecology and Obstetrics) [5]  - Norway  - 2020 | - Mainly based on the Norwegian Directorate of Health guideline, which in turn provides detailed information on selection process and grading of evidence | - Fasting blood glucose ≥5.3 mmol/l – 6.9 mmol/l and/or 2-hour 75g OGTT level ≥9.0mmol/l – 11.0 mmol/l | - HbA1c 4 months postpartum and then annual | - In addition to diabetes screening, a systematic follow-up – based on flow-chart for women with previous hypertensive pregnancy complications – to optimize cardiovascular health is recommended. This includes cardiovascular risk assessment and lifestyle advice with GP 6-12 weeks and 1 year postpartum, and then every 5 years until age 50, and medical intervention (e.g. for hypertension) according to national guidelines |
| - International Federation of Gynecology and Obstetrics [6]  - N/A  - 2015 | - Scientific literature, details of search not provided.  Quality assessment and grading of strength of recommendations provided | - IADPSG/WHO-criteria, based on 75g OGTT:  -Fasting plasma glucose 5.1−6.9 mmol/L (92−125 mg/dl)  - 1-hour value ≥10 mmol/L (180 mg/dl)  - 2-hour value 8.5–11.0 mmol/l  (153−199 mg/dl) | - 75g OGTT 6-12 weeks postpartum | - Irrespective of results of postpartum screening, women with previous GDM should be considered to have same or higher level of diabetes and CVD as people with prediabetes, and thus be given lifestyle advice to reduce this risk  - No concrete advice on how to follow-up, but health care providers encouraged to link check-ups for the child to the follow-up of the mother |

Additional file 4– Findings from the guidelines (continued)

| Name of society/organization  Country  Year published | Basis for recommendations | Definition of GDM | Recommendations for postpartum testing for diabetes | Recommendations for follow-up regarding cardiovascular risk |
| --- | --- | --- | --- | --- |
| - National Institute for Health and Care Excellence [7]  - UK  - 2015 | - Systematic literature search.  Stakeholder organizations also invited to submit evidence for consideration.  - Quality assessment and grading of strength of recommendations provided | - Fasting plasma glucose level ≥5.6 mmol/l or 2-hour 75 OGTT level ≥7.8 mmol/l | **-** Blood glucose should be tested before transferring to community care postpartum  - Fasting plasma glucose 6-13 weeks postpartum.  - Annual HbA1c for those with a negative postnatal test. | - CVD risk not addressed.  - Lifestyle advice for preventing T2DM suggested |
| - Socialstyrelsen (Swedish National Board of Health and Welfare) [8]  - Sweden  - 2018 | Literature search, details provided in supplementary material of the guideline | - Provided in reference: Based on 75g OGTT:  -Fasting blood glucose ≥5,1 mmol/l  - 1-hour ≥ 10,0 mmol/l  - 2-hour ≥ 8,5 mmol/l | - No specific recommendations, but general advice on following up blood glucose levels | - Health services should offer support for changing unhealthy lifestyle habits, and systematically follow-up weight blood glucose and cardiovascular risk factors  - Exact nature of this follow-up not provided |

# References

1. American College of Obstetricians and Gynecologists. ACOG Practice Bulletin No. 190: Gestational Diabetes Mellitus. 2018;131(2):e49-e64.

2. Dansk Selskab for Obstetrik og Gynækologi. Kliniske retningslinier for gestationel diabetes mellitus (GDM). Screening, diagnostik, behandling og kontrol samt follow-up efter fødslen København2010 [Available from: <https://endocrinology.dk/kliniske%20retningslinier%20-%20GDM.pdf>.

3. Deutsche Diabetes Gesellschaft; Deutsche Gesellschaft für Gynäkologie und Geburtshilfe. S3-Leitlinie Gestationsdiabetes mellitus (GDM), Diagnostik, Therapie und Nachsorge 2018 [Available from: <https://register.awmf.org/assets/guidelines/057-008l_S3_Gestationsdiabetes-mellitus-GDM-Diagnostik-Therapie-Nachsorge_2019-06.pdf>.

4. Feig DS, Berger H, Donovan L, Godbout A, Kader T, Keely E, et al. Diabetes and Pregnancy. Canadian journal of diabetes. 2018;42 Suppl 1:S255-s82.

5. Friis CMR, Ellen Marie Strøm; Holm, Helene Oeding; Toft, Johanne Holm; Roland, Marie Cecilie Paasche; Thordarson, Hrafnkell Baldur. Svangerskapsdiabetes Oslo: Norsk gynekologisk forening; 2020 [updated 16.02.2020. Available from: <https://www.legeforeningen.no/foreningsledd/fagmed/norsk-gynekologisk-forening/veiledere/veileder-i-fodselshjelp/svangerskapsdiabetes/>.

6. Hod M, Kapur A, Sacks DA, Hadar E, Agarwal M, Di Renzo GC, et al. The International Federation of Gynecology and Obstetrics (FIGO) Initiative on gestational diabetes mellitus: A pragmatic guide for diagnosis, management, and care. International journal of gynaecology and obstetrics: the official organ of the International Federation of Gynaecology and Obstetrics. 2015;131 Suppl 3:S173-211.

7. National Institute for Health and Care Excellence. Diabetes in pregnancy: management from preconception to the postnatal period London: NICE; 2015 [updated 16.12.2020. Available from: <https://www.nice.org.uk/guidance/ng3>.

8. Socialstyrelsen. Nationella riktlinjer för diabetesvård 2018 [Available from: <https://www.socialstyrelsen.se/globalassets/sharepoint-dokument/artikelkatalog/nationella-riktlinjer/2018-10-25.pdf>.
